# Artificial Intelligence on Diagnostic Aid of Leprosy: A Systematic Literature Review

**DOI:** 10.3390/jcm13010180

**Published:** 2023-12-28

**Authors:** Jacks Renan Neves Fernandes, Ariel Soares Teles, Thayaná Ribeiro Silva Fernandes, Lucas Daniel Batista Lima, Surjeet Balhara, Nishu Gupta, Silmar Teixeira

**Affiliations:** 1 PhD Program in Biotechnology—Northeast Biotechnology Network, Federal University of Piauí, Teresina 64049-550, Brazil; jacks.renan@ifpi.edu.br; 2 Postgraduate Program in Biotechnology, Parnaíba Delta Federal University, Parnaíba 64202-020, Brazil; thayana.fernandes@hotmail.com (T.R.S.F.); lucas.daniel.bp@gmail.com (L.D.B.L.); silmarteixeira@ufpi.edu.br (S.T.); 3 Federal Institute of Maranhão, Araioses 65570-000, Brazil; 4 Department of Electronics & Communication Engineering, Bharati Vidyapeeth’s College of Engineering, New Delhi 110063, India; surjeet.balhara@bharatividyapeeth.edu; 5 Department of Electronic Systems, Faculty of Information Technology and Electrical Engineering, Norwegian University of Science and Technology, 2815 Gjøvik, Norway; nishugupta@ieee.org

**Keywords:** Hansen’s disease, leprosy, diagnosis, artificial intelligence, deep learning, machine learning

## Abstract

Leprosy is a neglected tropical disease that can cause physical injury and mental disability. Diagnosis is primarily clinical, but can be inconclusive due to the absence of initial symptoms and similarity to other dermatological diseases. Artificial intelligence (AI) techniques have been used in dermatology, assisting clinical procedures and diagnostics. In particular, AI-supported solutions have been proposed in the literature to aid in the diagnosis of leprosy, and this Systematic Literature Review (SLR) aims to characterize the state of the art. This SLR followed the preferred reporting items for systematic reviews and meta-analyses (PRISMA) framework and was conducted in the following databases: ACM Digital Library, IEEE Digital Library, ISI Web of Science, Scopus, and PubMed. Potentially relevant research articles were retrieved. The researchers applied criteria to select the studies, assess their quality, and perform the data extraction process. Moreover, 1659 studies were retrieved, of which 21 were included in the review after selection. Most of the studies used images of skin lesions, classical machine learning algorithms, and multi-class classification tasks to develop models to diagnose dermatological diseases. Most of the reviewed articles did not target leprosy as the study’s primary objective but rather the classification of different skin diseases (among them, leprosy). Although AI-supported leprosy diagnosis is constantly evolving, research in this area is still in its early stage, then studies are required to make AI solutions mature enough to be transformed into clinical practice. Expanding research efforts on leprosy diagnosis, coupled with the advocacy of open science in leveraging AI for diagnostic support, can yield robust and influential outcomes.

## 1. Introduction

Neglected tropical diseases (NTDs) can compromise people’s quality of life, leading to physical and psychological disabilities. Such diseases are caused by infectious agents or parasites and affect more than one billion people worldwide [1]. They are more prevalent in populations in Latin America, Africa, and Asia [2,3], and considered endemic in 13 low- and middle-income countries [4]. Leprosy is a NTD and considered one of the oldest diseases in human history [5]. The infectious agent of the disease is the intracellular parasite *Mycobacterium leprae* (*M. leprae*) or Hansen’s bacillus, which may affect the skin, peripheral nerves, eyes, endothelial cells, bones, mucous membranes, and may result in physical injuries and mental disabilities [6,7]. Approximately 210,000 new cases are reported annually, with 15,000 cases identified in children [8]. Leprosy is present in more than 150 countries, with 80% of cases concentrated in India, Brazil and Indonesia, and considered a public health problem [1].

Despite its high incidence in some regions, estimates show that only 5% of people exposed to the leprosy pathogen are actually infected, and only 20% of them develop the disease [9]. Even people who do not develop the disease, because they have innate immunity, may experience a period in which the bacillus is released through the upper respiratory tract, which is the most common route of transmission [10,11]. Diagnosing leprosy is a challenging task since the symptoms take from two months to 20 years to appear, and there is no gold standard test to diagnose it [12]. The diagnosis is predominantly made by analyzing clinical and dermato-neurological signs, and complementary tests such as heat sensitivity test and Mitsuda intradermal reaction test, and serology may also be used [13]. However, the absence of early symptomatology and similarity to other dermatological conditions can lead to an inconclusive diagnosis and, consequently, lack of appropriate treatment [14].

Artificial intelligence (AI) technology is a growing area and thanks to machine/deep learning (ML/DL), it has gained increasing prominence in medicine. ML/DL techniques encompass statistical models and algorithms capable of progressively learning from data, predicting features, and executing a task [15]. In particular, DL systems can process complex, high-dimensional data such as images [16,17]. In recent years, ML/DL applications have increased exponentially as a diagnostic aid in dermatology [18,19]. Methods for the analysis and classification of dermatological lesions may involve steps such as image acquisition, pre-processing, segmentation, feature extraction and lesion classification [5,20].

In recent years, secondary studies have addressed the use of AI in the health area, especially in dermatology, given the need for recognition and analysis of images with high speed and accuracy [21]. For example, Brinker et al. [22] reviewed studies focused on the development of skin lesion classifiers using Convolutional Neural Networks (CNNs). Popescu et al. [23] presented the advances in the detection of melanoma using artificial neural networks (ANNs). Wu et al. [24] provided an overview of the algorithms based on DL for skin cancer classification, while Kumar et al. [25] gathered data related to AI techniques for diagnosing various diseases, including skin diseases. Yu et al. [26] summarized a set of ML applications for psoriasis assessment and management. Different from the previous secondary studies, this Systematic Literature Review (SLR) aims to identify, analyze and characterize the state-of-the-art AI techniques for diagnostic aid of leprosy.

The remaining article is organized as follows. Section 2 addresses relevant concepts related to leprosy and its diagnosis methods, as well as concepts and application of AI in clinical medicine, for a better understanding of the review. Section 3 describes the research methodology. In Section 4, the selected studies are detailed to answer the research questions, while we discuss trends, open issues and limitations in Section 5. Finally, Section 6 concludes the review.

## 2. Background

### 2.1. Leprosy

Leprosy, one of humanity’s oldest diseases, remains a significant public health problem worldwide despite being treatable [7]. This chronic infectious condition, caused by Mycobacterium leprae, can affect the cells lining blood and lymph vessels, sensory, motor and autonomic nerves, eyes, bones, and the upper respiratory tract [27,28]. Exploring the epidemiology of this disease unveils the complex dynamics underlying its occurrence [9]. In addition to the medical complexities, individuals diagnosed with leprosy endure social discrimination, face social exclusion, suffer from a diminished quality of life and often struggle with permanent disfigurement [29].

About the transmissibility, even people who do not develop the condition may experience a period in which the bacillus is released through the upper respiratory tract, which is the most common route of transmission [10,11]. This transmission occurs through close and prolonged contact between a susceptible individual and an infected bacillus. In addition, less common transmission can also occur through skin erosion and vertical transmission. Thus, an infected bacillus carrier is essential in transmitting leprosy [30,31,32]. The infected individual who develops the disease may present characteristic symptoms that can determine the type of classification and, consequently, the treatment.

According to the World Health Organization (WHO), leprosy can be categorized as paucibacillary (PB) or multibacillary (MB), depending on the individual’s immune response to *M. leprae*. This classification is called Operational and guides the appropriate therapeutic regimen for the patient, designated for treatment purposes. In addition, it is based on the clinical appearance and bacterial index of the lesions. Individuals who have up to five skin lesions and negative intradermal smears are considered PB, and those with six or more skin lesions and positive intradermal smears are classified as MB [33,34].

*M. leprae* leads to loss of sensation, innervation, damage within the epidermis, and lesions, which are associated with loss of myelin in Schwann cells. In Brazil, one of the most endemic countries for the disease, the Madrid Classification (1953) [35] is used, which was later adapted by Ridley and Jopling (1966) and is widely used throughout the world. The Madrid classification also determines the type of leprosy according to the characteristics of the lesions, neural involvement and sensitivity. It subdivides them into indeterminate, tuberculoid, borderline and virchowian leprosy and is widely used for the differential diagnosis of leprosy [36,37]. Ridley and Jopling [38] classified leprosy into five clinical forms based on clinical, histopathological, immunological, and bacilloscopic characteristics: tuberculoid–tuberculoid, borderline-tuberculoid, borderline–borderline, borderline-lepromatous, and lepromatous–lepromatous.

### 2.2. Diagnosis Methods of Leprosy

Approximately 70% of leprosy cases are clinically diagnosed through clinical and epidemiological history, anamnesis, dermatological and neurological evaluation [39]. Clinical diagnosis is based on three cardinal signs: (1) definite loss of sensation in a hypopigmented or reddened skin patch; (2) peripheral nerve thickening, with loss of sensation and weakness of the muscles innervated by the affected nerve; and (3) microscopic detection of bacilli [1,40,41]. However, 30% of patients do not have the typical characteristics of the disease, requiring additional tests, such as the Mitsuda reaction test, serological tests, and molecular biology tests [37,42]. Specifically, on the clinical diagnosis performed by health professionals, the patient’s dermatological and neurological signs and symptoms are evaluated. Skin lesions are identified when present, and a thermal sensitivity test is performed to assess sensitivity changes in the lesions [27,37].

The neurological field also needs to be assessed. Tests for analysis of irritation or itching in the eyes and bleeding or wounds in the nose, palpation of peripheral nerve trunks, and assessment of muscle strength and joint mobility in hands and feet are necessary to verify neural involvement [39,43,44]. Hand and foot mobility may be assessed by the Graded Sensory Test [45]. Neuropathic pain affects more than 60% of leprosy patients and is caused by primary damage to fine fibers, unmyelinated fibers or dysfunction of the nervous system, and can be assessed by electroneuromyography [46].

The clinical diagnosis is often insufficient, requiring additional tests, such as laboratory tests [37,41]. Bacilloscopy, for example, allows the detection of alcohol-acid-resistant bacilli, such as Hansen’s bacillus, and has a specificity of 100% and a sensitivity that varies between 34.4% and 50%. For its application, it is necessary to collect smears of intradermal scrapings from regions such as right and left earlobes, right and left elbows, and skin lesions [47,48]. Another critical test is the histopathological one, which is performed using samples from the edges of more active lesions and nerves that can help diagnose cases with atypical clinical manifestations and then direct a more accurate treatment. The specificity of this exam is from 70% to 72%; on the other hand, its sensitivity is low, ranging from 49% to 70% [33,48,49].

The Mitsuda intradermal reaction test is a skin reaction based on an individual response of high sensitivity and high specificity of the delayed cell type against the bacillus *M. leprae*. Fernández’s intradermal reaction test, on the other hand, has an early reaction with low sensitivity, thus presenting a risk of cross-reactivity with other bacteria. Therefore, the Mitsuda test is the most widely used [37]. Positive indicates that an individual’s macrophages can destroy the bacillus *M. leprae* and, when negative, if exposed to *M. leprae*, he is at greater risk of becoming ill and developing the virchowian form of leprosy. In addition, the test helps to classify leprosy as undefined and borderline [50].

Another widely used test is serological, which is important for evaluating and quantifying the bacterial load of *M. leprae*. Phenolic glycolipid-I (PGL-1) is the major antigenic glycolipid of *M. leprae* and allows the detection of anti-PGL-1 immunoglobulin G (IgG) and immunoglobulin M (IgM) antibodies. The presence of IgM antibodies in response to PGL-I, which is present in the cell wall of *M. leprae*, helps to classify leprosy (low bacterial load for PB and high bacterial load for MB). Among the numerous methodologies, two are widely used: the enzyme-linked immunosorbent assay (ELISA) and ML-Flow, an alternative method to ELISA but with a lateral flow format [36]. In addition, serology is essential to identify household contacts at higher risk of developing the disease, as well as in the follow-up of cases to assess the risk of relapse [36,51].

One of the most specific diagnostic methods is those based on molecular tests, which are based on species-specific sequences, deoxyribonucleic acid (DNA) and ribonucleic acid (RNA), such as the polymerase chain reaction (PCR) [47,52]. This methodology presents high specificity and sensitivity due to the success in detecting Hansen’s bacillus DNA, even when there are few [53,54,55].

### 2.3. Artificial Intelligence in Clinical Medicine

AI refers to the ability of intelligent agents to learn and solve problems in automated processes that impact the quality of life in society, from the automation of industrial processes and communication by smartphones to diagnostic help in medicine [21]. Machine Learning (ML) is a subfield of AI, which uses autonomous algorithms and statistical models that identify patterns and have the potential to help diagnose diseases and other medical approaches [56]. Deep learning (DL) is a sub-area of ML and uses the concept of artificial neuron layers for pattern extraction, and representation of complex and unstructured data [57,58].

Computer vision (CV), combined with IA models, enables the analysis of medical images, assisting in the diagnostic process and potentially reducing human errors [59]. Some diagnostic models based on CV have shown significant evidence in improving the detection of diseases, such as in the early diagnosis aid of skin cancer [60].

In healthcare, AI can provide suggestions and recommendations that direct the decision-making process in clinical practice, facilitated by evaluation and testing, notwithstanding barriers, such as data availability and quality [61,62,63]. Disease prediction models use AI techniques (i.e., ML/DL) associated with data mining approaches [64]. Cardiology, pulmonary medicine, endocrinology, nephrology, gastroenterology, and neurology are some application areas of AI in the medical practice [65,66].

The diagnostic method involves detecting a disease or health condition by analyzing the individual’s clinical signs and symptoms. AI models have been used to facilitate the diagnostic process, with the development of methods capable of analyzing, classifying, and predicting an outcome using a dataset related to the various existing pathologies, such as cancer, diabetes, dengue, malaria, tuberculosis [17], and mental health [67,68].

Moreover, improvements in the health area from AI have also reached the dermatological field [69]. ML techniques are helpful in diagnosis through skin image analysis, and a future trend in this area regarding personalized treatment [21,70,71]. Advances are not restricted to the analysis of melanomas and pigmented skin lesions. Other dermatological conditions are analyzed, such as psoriasis, acne, autoimmune disorders, and allergic contact dermatitis [20]. Promising results have been demonstrated in the detection of monkeypox lesions using the MobileNetV2 architecture [72], in the early detection of skin cancer [15], in which CNNs show high accuracy in disease recognition [73].

## 3. Methodology

This SLR followed the preferred reporting items for systematic reviews and meta-analyses (PRISMA) framework [74] (see PRISMA checklist in Supplementary File S1). We addressed three distinct phases: (i) planning the conduction of the review by elaborating a review protocol; (ii) performing collaboratively the activities contained in the protocol using the online tool *Parsif.al* [75]; and (iii) extracting data from the selected articles, analyzing and synthesizing the relevant information on the research topic. We registered the review protocol on PROSPERO (registration number CRD42023400323).

### 3.1. Research Questions

The following Research Questions (RQs) were defined:

- (RQ1) What types of leprosy are targeted by AI models?
- (RQ2) What data types were used to develop AI models?
- (RQ3) What preprocessing techniques were used on the datasets?
- (RQ4) What AI algorithms/architectures were applied to diagnose leprosy?
- (RQ5) How well do the models perform?

### 3.2. Search Strategy and Selection Criteria

The following digital libraries were used to search for primary studies: ACM Digital Library, IEEE Digital Library, Web of Science, Scopus, and PubMed. The search process, conducted by one researcher, occurred on 31 August 2023; it was verified by two other researchers. To retrieve relevant studies, we used two search terms combined with their synonyms to design the search string presented in Box 1. The following three control articles were selected to guide the searches in the digital libraries [12,14,76]. The validation process of the search strings occurred in the databases, which demonstrated their ability to find studies suitable for this SLR, including the control articles.

Box 1 **Search string used for this SLR.**

“((“Artificial Intelligence” OR “Data Science” OR “Deep Learning” OR “Machine Learning” OR “Algorithm*” OR “Predict* Model*” OR “Big Data” OR “Transfer Learning” OR “Computer Vision” OR “Text Mining” OR “Dataset” OR “Support Vector Machine” OR “Artificial Neural Network” OR “Backpropagation Neural Network” OR “Convolutional Neural Network” OR “Neural network” OR “Pattern recognition” OR “Supervised Learning” OR “Generative Adversarial Network” OR “Feature Learning” OR “Meta Data” OR “Image Segmentation” OR “Image Classifiers” OR “Image Processing” OR “Fuzzy Logic” OR “Decision Tree” OR “Decision Support System” OR “Support Vector Regression” OR “Regression” OR “Bayesian” OR “K-nearest Neighbors” OR “K-means”) AND (“Leprosy” OR “Hansen’s Disease”))”.

Inclusion and exclusion criteria were defined for selecting articles, as listed in Table 1. Initially, we retrieved documents and compared them to remove duplicate records. We screened articles for eligibility based on their title, abstract, and keywords. In a second moment, the researchers read and analyzed the full text of the screened studies to identify those suitable for the scope of this review. We then evaluated the selection process by applying Cohen’s Kappa coefficient [77], which measures the level of agreement between researchers’ analyses. In the end, when there was no consensus among the researchers, the other researchers (co-authors) held discussions to resolve selection conflicts. We finally performed the snowballing technique [78,79] to maximize results in the selection process.

### 3.3. Quality Assessment

Two independent researchers evaluated the selected studies using a quality assessment tool adapted by Cabitza and Campagner [80] to evaluate the robustness of the methodology in medical machine learning studies and the ability to reproduce its findings qualitatively. The checklist contains 30 items, which are quality criteria (QC), organized into six phases: problem understanding, data understanding, data preparation, modeling, validation, and deployment. Each item represents a requirement and is associated with three possible options: adequately addressed (OK), sufficient but unlikely, minor revision required (mR), and inadequately addressed, major revision required (MR). The studies were individually classified on a trichotomous scale associated with the tool’s options, with a score of 1 (OK), 0.5 (mR), and 0 (MR). The quality assessment score is calculated based on the sum of the scores assigned to the items. Two researchers who analyzed the studies to assign the scores resolved evaluation conflicts through discussions. When there was no agreement, a third researcher acted as a judge and resolved the conflicts. The QCs used are shown below in Table 2.

### 3.4. Data Extraction

The researchers read each selected article to extract the necessary information to answer the research questions, characterize the studies, and outline opportunities for future work. Table 3 presents the items of the data extraction form and their respective research question.

## 4. Results

### 4.1. Study Selection

**Figure 1:**
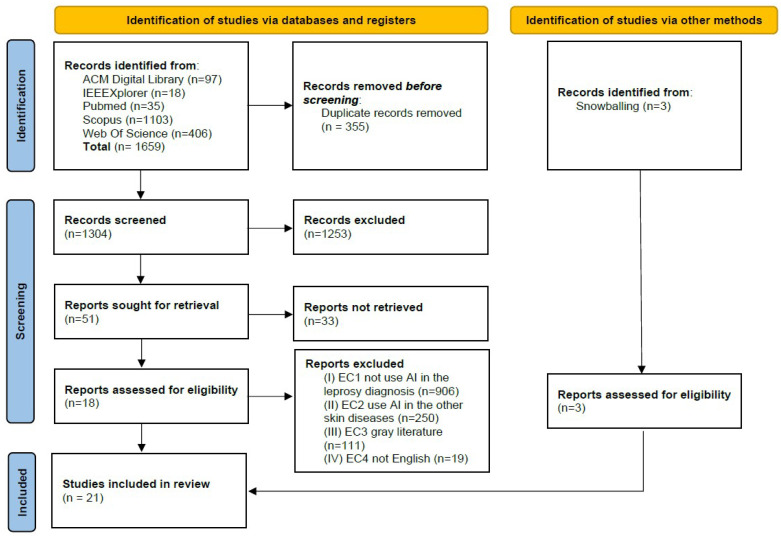
PRISMA flow diagram.

Figure 1 presents the PRISMA flow diagram with the study selection process. We initially retrieved 1659 articles from digital libraries. We then identified and removed 355 duplicate studies. Among the remaining 1304 articles, 51 were selected by reading the title and abstract. Eighteen papers were eligible after a complete reading of the study. Cohen’s Kappa test between researchers’ analyses was Kappa = 0.84 (*p* < 0.001), considered “almost perfect agreement” [81]. We resolved conflicts through discussions, resulting in 18 studies. The snowballing technique resulted in the addition of three studies meeting the selection criteria. A total of 21 studies were included in the review for qualitative analysis.

### 4.2. Study Characterization

Table 4 presents the data extracted from the selected articles to answer the RQs.

### 4.3. Answering the Research Questions

#### 4.3.1. Leprosy Types Targeted by AI Models (RQ1)

No study classified the types of leprosy according to the Madrid classification or Ridley Jopling classification. Some papers have classified leprosy according to the operational classification, which is recommended by the WHO. Studies by [14,87,94] classified leprosy as either paucibacillary or multibacillary. Binary classification occurred in 43% of the selected articles [12,14,76,82,83,87,94,95,96]. The remaining 57% of the articles used multiclass classification tasks, in which the models classified different skin diseases and, among them, leprosy [84,85,86,88,89,90,91,92,93,97,98,99]. Also, results revealed that 24% were classified as leprosy or not. Most papers do not prioritize leprosy in proposing an AI model to aid in diagnosing the condition. Therefore, the works are directed at classifying skin diseases, including leprosy.

#### 4.3.2. Data Types (RQ2)

The data types used in most models were images of skin lesions, and models classified leprosy against other dermatological diseases [12,82,83,84,85,86,88,89,90,91,92,93,97,98]. However, Barbieri et al. [12] used images of skin lesions combined with clinical information to develop an AI model. The clinical information was the loss of thermal sensation, nodules and papules, paresthesia in the feet, number of lesions, sex, flaking surface, itching, trunk, absence of symptoms in the skin lesion, and diffuse infiltration.

The studies [14,76,87,94,96] utilized the outcomes of tests as input data to AI models, such as the RNA sequencing technique (RNA-Seq) and real-time reverse transcription polymerase chain reaction (RT-qPCR) used in molecular and cellular biology. The RNA-Seq technique extracts total RNA from a biological sample, converts it into complementary DNA (cDNA), and performs next-generation sequencing (NGS) [100]. The RT-qPCR and RNA-Seq techniques are used to quantify gene expression [101]. The study by Tió-Coma et al. [76] used the results of gene expression analyses from RNA-Seq and RT-qPCR as input to develop an AI model. The study by Pillai and Chouhan [96] analyzed the H37Rv strain to study the immunology and pathogenesis of tuberculosis. H37Rv is a strain of Mycobacterium tuberculosis [102] and share characteristics similar to *M. leprae*.

To create an AI model, the study by Gama et al. [94] used the data age, sex, treatment time, qPCR test result (*M. leprae* DNA level), IgG/IgM serology level, and sputum smear index. The IgG/IgM serology levels tell about the amount of IgG/IgM antibodies present in a person’s blood sample and indicate whether the person has been exposed to a pathogen, virus, or bacteria [103]. The sputum smear index is an indicator to assess the bacillary load of a Mycobacterium in a sputum sample [104].

Cytokines are a group of signaling molecules produced by the immune system, such as tumor necrosis factor (TNF), interferon-gamma (IFN-y), interleukin 4 (IL-4), and interleukin 10 (IL-10), and their presence can indicate a specific disease [105]. Marçal et al. [14] used results from an in vitro assay model of the *M. leprae* antigen and measurements of the cytokines TNF, IFN-y, IL-4, and IL-10 as input to develop an AI model for the operational classification of leprosy.

#### 4.3.3. Preprocessing Techniques (RQ3)

Preprocessing techniques to prepare the dataset may depend on the choice of data type and the algorithm or architecture used to train the model. Some authors used numerical data, so requiring the data to be normalized [12]. Researchers who used datasets with images and ML classical algorithms in [86,89,92,93,97,98,99] had to apply various image preparation techniques and feature extraction. The features most explored by the authors were related to texture and edges, with applications of spatial filters aiming to correct, smooth, or enhance specific regions. In addition, image compression techniques (e.g., YCbCr algorithm [97] and DCT [89,98]), segmentation-related techniques (e.g., binary mask [92,97], histogram [90,97], OTSU [86], global thresholding [93]), and image noise reduction techniques (e.g., median filter, smooth filter [92,97]) were explored by the studies. Jin et al. [90] used the ResNet-50 and VGG16 architectures and the HOG technique for feature extraction. Mondal et al. [85,91] used techniques for image normalization and augmentation; [83,84] used data augmentation.

#### 4.3.4. Algorithms and Architectures (RQ4)

Most of the authors chose classical ML techniques. Researchers in [12,14,76,87,94] used the classical algorithms MLP, LR, RF, and DT to train numerical datasets. Researchers in [86,89,90,92,93,97,98,99] utilized classic ML algorithms DT, SVM, FFBPN, and ANN to build models with image datasets. Researchers in [82,83,84,85,88,91,95] used the MobileNet-V2, DenseNet-121, Inception-V3, ResNet-50, EfficientNet-B2, LeprosyNet, and Siamese Network architectures to develop models with image datasets. Refer to the references [106,107] for a comprehensive understanding of the ML algorithms and ANN architectures utilized in the studies.

#### 4.3.5. Performance of the Models (RQ5)

The most common metrics used to measure the performance of AI models were found in the selected papers: accuracy, precision, sensitivity/recall, specificity, F1 score, and AUC. The review revealed that accuracy is the metric used by the authors in 90% of the studies. In addition, accuracy was the only metric to measure model performance in 52% of articles [14,82,85,89,90,93,95,96,97,98,99]. Accuracy can be misleading in multiclass classification tasks with imbalanced datasets, in which one class may have more samples than others. In such cases, other metrics, such as precision, recall, and F1 score, should be utilized together with accuracy for better clarity of performance [108].

**Figure 2:**
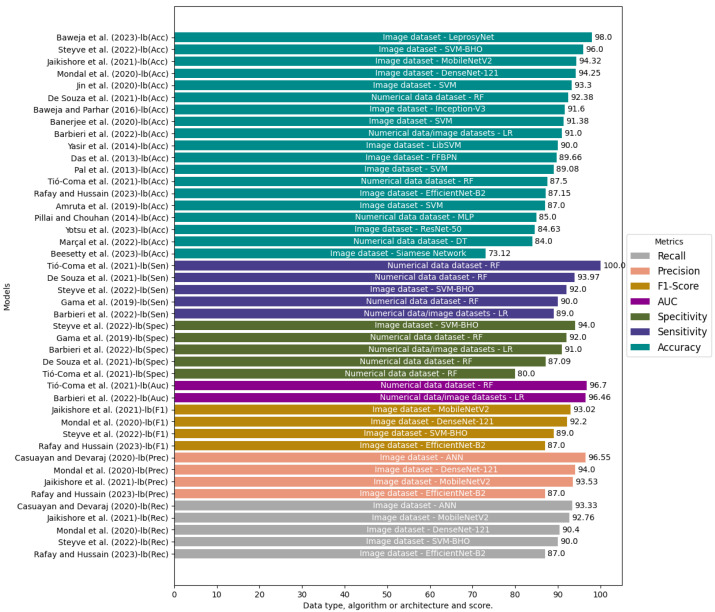
Data types, algorithms/architectures, performance metrics and results of the selected studies [12,14,76,82,83,84,85,86,87,88,89,90,91,92,93,94,95,96,97,98,99].

Figure 2 shows the metrics, the data types, the algorithms/architectures, and the performance of the models developed by the selected studies. A study implementing an architecture with a binary classification task called LeprosyNet obtained the best performance when considering accuracy. The most used algorithm among the selected studies is the SVM. In addition, the research revealed that the best DL techniques that used multiclass classification tasks of leprosy against other dermatoses were the CNN MobileNet-V2 and DenseNet-121 architectures. The models developed from image datasets had an average accuracy in the classification of leprosy of 89.97%. In comparison, the models created from numerical datasets reached an average accuracy of 87.98%.

### 4.4. Study Quality

**Figure 3:**
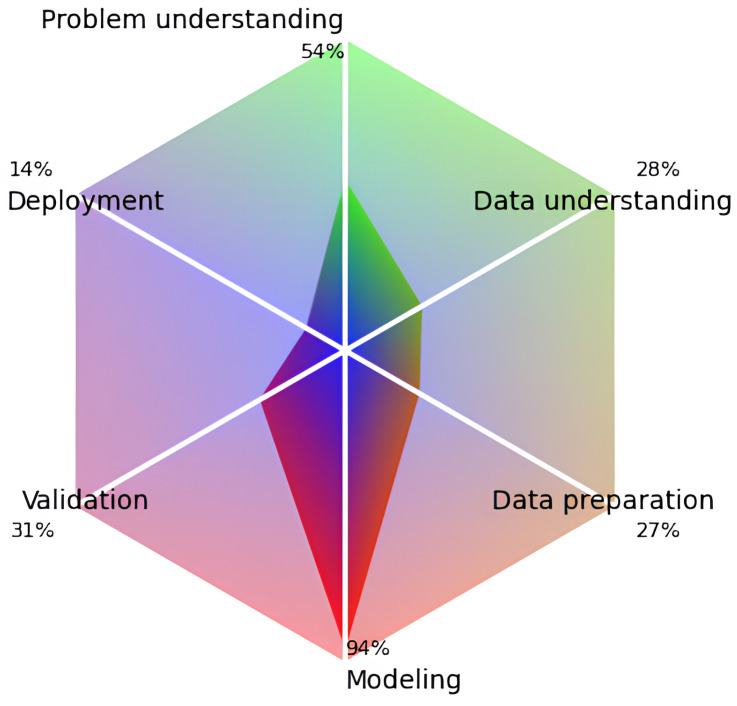
Quality assessment results for the six phases of the checklist [80].

Evaluating the quality of the selected studies allowed us to qualitatively analyze the methodological rigor of the ML studies in the medical area, their contributions, and the reproducibility of the results (see detailed quality assessment per study in Supplementary File S2). In 11 articles [83,86,90,92,93,94,95,96,97,98,99], the scores reached less than 40% of the tool score. Other studies attained results above 40% of the quality criteria [76,82,87], and the study in [12] achieved 63.3%, which was the best evaluation. Figure 3 depicts an overview of the quality assessment result per phase of the checklist.

The modeling phase was adequately addressed by all studies evaluated. In the problem-understanding phase, the studies in [12,14,76,82,85,87,88,89,90,94,97] demonstrated satisfactory quality. In contrast, the remaining studies could have provided more robust information in the problem-understanding phase. In the phases of data understanding, data preparation, validation and deployment, the studies failed to address the quality criteria assessed.

## 5. Discussion

To the best of our knowledge, this is the first SLR to focus on leprosy diagnostic aid supported by AI techniques. Therefore, this work can help researchers in AI and health informatics by characterizing the studies regarding datasets, preprocessing techniques, AI algorithms/architectures, also comparing the performance of different ML/DL models. In this section, we identify trends and open issues in current research, which are opportunities for future research. Also, we acknowledge the limitations of this SLR.

### 5.1. Trends

We recognized several trends presented by the studies on the diagnostic aid of AI-supported leprosy. First, we identified a trend of using images in datasets (n = 16) for developing AI models for leprosy classification. We also recognized a trend to use classical supervised ML algorithms (n = 14), highlighting SVM (n = 8), RF (n = 5) and DT (n = 4) as the most used ones. We also recognize that most models are developed for multiclass classification tasks (n = 12), and the metric most used was accuracy (n = 19).

**Figure 4:**
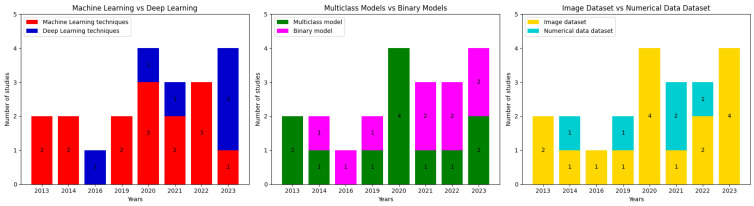
Number of articles published by year, organized according to different characteristics.

Figure 4 displays three bar charts with the number of papers published by year, categorized by the type of model (i.e., classical machine learning vs. deep learning), the type of task modeled (i.e., multiclass classification task vs. binary classification task), and the type of dataset (image data vs. numerical data).

### 5.2. Open Issues

Studies identified in our SLR present promising solutions for diagnostic aid of leprosy using AI techniques. However, we recognize that there are open issues to be addressed by further research.

#### 5.2.1. Open Science

Open science promotes openness and accessibility of research results, including data and methods. Reproducibility is an essential component of open science, as it aims to ensure that the results of a study can be reproduced and validated by other researchers [80]. Open science adopts and promotes the Findable, Accessible, Interoperable, Reusable (FAIR) principles, which are guidelines that aim to make scientific data auditable [109]. In this regard, accountability in AI governance ensures that research is conducted ethically, transparently, and responsibly [110].

In most studies (n = 17) identified in our SLR, the dataset, code, and methods used to implement AI models were not shared in public repositories, which impacted the quality assessment of the studies (see Section 4.4). Thus, this open issue can (and should) be addressed, such as implementing AI models that can aid in leprosy diagnosis following the principles of open science by sharing work information in a public repository under a permissive license to undergo external validation. This is enabled by free online repository services, such as GitHub and Zenodo.

#### 5.2.2. Data Fusion

Data fusion in AI refers to combining and integrating information from various data sources that may include different types of data, such as text, images, audio, and databases [111]. Data fusion aims to leverage the complementary information from each data source to improve the accuracy, reliability, and understanding of the results obtained. The fusion process can involve data integration, alignment, aggregation techniques, and the application of ML algorithms to explore and extract knowledge from the combined data [112].

Barbiere et al. [12] combined skin lesion images with clinical data from leprosy patients to train disease classification models. An open issue is an in-depth exploration of the combination of different multimodal data and originated from different sources (e.g., personal information, clinical signs and symptoms, skin lesion images, and information on reactions to polychemotherapy) to implement AI models to contribute to the diagnosis of leprosy.

#### 5.2.3. Differential Diagnostic

Several diseases have skin lesions with characteristics similar to leprosy, which can significantly increase the rate of false diagnosis, hence the stigma associated with the disease [113]. The results revealed that proposed AI models classified leprosy according to different classification forms (e.g., paucibacillary vs. multibacillary; binary classification to identify the presence of leprosy; and leprosy classified against other skin diseases). Yet, a research gap to be addressed is to build AI models through image analysis to classify the skin lesions caused by leprosy according to its clinical forms (e.g., Madrid or Ridley and Jopling classifications), so facilitating the differential diagnosis; that is, the distinction between leprosy and other dermatological conditions that may present similar symptoms.

#### 5.2.4. External Validation

External validation refers to testing a model’s ability to make accurate and useful predictions on datasets not used during training, and then providing evidence of the model’s generalizability. External validation can improve the reliability of models, allowing them to be applied safely in different populations and clinical environments [114,115,116]. None of the studies identified in our review externally validate their developed models (see detailed quality assessment per study in Supplementary File S2). Therefore, this remains an open issue that needs attention in future studies to ensure the prediction models’ reliability and utility.

### 5.3. Limitations of the SLR

This SLR has limitations to be acknowledged and considered when conducting future research. First, we did not review gray literature, so we did not include articles such as research reports, theses, dissertations, government reports, and tutorials. Consequently, future work may extend this SLR to consider gray literature on this research topic. Second, we searched for articles only in the leading digital libraries. Therefore, future work may also extend the search to additional databases.

## 6. Conclusions

The results of this SLR provided new insights into the literature related to AI techniques in aiding leprosy diagnosis. Key trends were identified, such as the prevalence of classical supervised ML algorithms, and that most models are developed for multiclass classification tasks and using dermatological images as a non-invasive technique. Most of the articles did not consider leprosy as the study’s primary objective but rather the classification of different skin diseases and, among them, leprosy. In addition, most of the selected papers did not adhere to the open science principles, showing low quality regarding transparency, data sharing, and responsibility. Such findings highlight the need for more research on leprosy diagnosis and to promote open science in the application of AI in healthcare to ensure reliable and impactful results. Therefore, AI-supported leprosy diagnosis is constantly evolving, but research in the area is still at an early stage, so it is not mature enough to be transformed into clinical practice.

## Figures and Tables

**Table 1:** Selection criteria.

Inclusion Criteria (IC)	Exclusion Criteria (EC)
(IC1) Studies that used AI to aid in the leprosy diagnosis.	(EC1) Studies that did not use AI in the leprosy diagnosis.
(IC2) Full articles.	(EC2) Studies that used AI to diagnose other skin diseases.
(IC3) Articles in English.	(EC3) Gray literature: reviews, reports, short papers, conference abstracts, communications, theses, and dissertations.
(IC4) Peer-reviewed articles.	(EC4) Articles in a language other than English.

**Table 2:** Quality criteria [80].

Item	Problem Understanding
(QC1)	Is the study population described, also in terms of inclusion/exclusion criteria?
(QC2)	Is the study design described?
(QC3)	Is the study setting described?
(QC4)	Is the source of data described?
(QC5)	Is the medical task reported??
(QC6)	Is the data collection process described, also in terms of setting-specific data collection strategies?
**Item**	**Data Understanding**
(QC7)	Are the subject demographics described in terms of average age, age variability, gender breakdown, main comorbidities, ethnic group, socioeconomic status?
(QC8)	If the task is supervised, is the gold standard described?
(QC9)	In the case of tabular data, are the features described?
**Item**	**Data Preparation**
(QC10)	Is outlier detection and analysis performed and reported?
(QC11)	Is missing-value management described?
(QC12)	Is feature pre-processing performed and described?
(QC13)	Is data imbalance analysis and adjustment performed and reported?
**Item**	**Modeling**
(QC14)	Is the model task reported?
(QC15)	Is the model output specified?
(QC16)	Is the model architecture or type described?
**Item**	**Validation**
(QC17)	Is the data splitting described (e.g., no data splitting; k-fold cross-validation (CV); nested k-fold CV; repeated CV; bootstrap validation; leave-one-out CV; 80%/10%10% train/validation/test)?
(QC18)	Are the model training and selection described?
(QC19)	(classification models) Is the model calibration described?
(QC20)	Is the internal/internal-external model validation procedure described, (e.g., internal 10-fold CV, time-based cross-validation)?
(QC21)	Has the model been externally validated?
(QC22)	Are the main error-based metrics used?
(QC23)	Are some relevant errors described?
**Item**	**Deployment**
(QC24)	Is the target user indicated?
(QC25)	(Classification models) Is the utility of the model discussed?
(QC26)	Is information regarding model interpretability and explainability available?
(QC27)	Is there any discussion regarding model fairness, ethical concerns, or bias risks, (for a list of clinically relevant biases, refer to)?
(QC28)	Is any point made about the environmental sustainability of the model, the carbon footprint, of either the training phase or inference phase (use) of the model?
(QC29)	Is code and data shared with the community?
(QC30)	Is the system already adopted in daily practice?

**Table 3:** Data extraction form.

Research Questions	Form Questions
(RQ1)	What types of leprosy were targeted?
(RQ2)	What data types are used in the dataset?
(RQ3)	What data preparation techniques?
(RQ3)	What was the data preparation process?
(RQ4)	What algorithm/architecture was used to develop models?
(RQ5)	How was the model evaluated?
(RQ5)	What performance metrics were used?
(RQ5)	How well did the models perform?

**Table 4:** Data extracted from the selected articles.

Study	Diseases	Data Types	Data Preparation	Algorithm/Architecture	Model Evaluation	Performance Metrics
Beesetty et al. (2023) [82]	Leprosy and other skin lesions	Images	Not Available	Siamese Network and Inception-V3, Adaptive Moment Estimation (ADAM)	Not Available	Accuracy (73.12%)
Baweja et al. (2023) [83]	Leprosy and other skin lesions	Images	Data augmented by Rotation, Scale Transformation, Blurring	AlexNet, ResNet, and LeprosyNet, optimized by ADAM	80/20	Accuracy (98.00%)
Rafay and Hussain (2023) [84]	Leprosy Borderline, Leprosy Lepromatous, Leprosy Tuberculoid, Basal Cell Carcinoma, Dariers’s Disease, Epidermolysis Bullosa Pruriginosa, Hailey-Hailey Disease, Herpes Simplex, Impetigo, Larva Migrans, Lichen Planus, Lupus, Melanoma, Molluscum Contagiosum, Mycosis Fungoides, Neurofibromatosis, Papilomatosis Confluentes And Reticulate, Pediculosis Capitis, Pityriases Rosea, Porokeratosis Actinic, Psoriasis, Tinea Corporis, Tinea Nigra, Tungiasis, Actinic Keratosis, Dermatofibrona, Nevus, Pigmented Benign Keratosis, Squamous Cell Carcinoma and Vascular Lesion	Images	Data augmented by Rotation, Shear, Center Zoom, Horizontal Flip, Vertical Flip, Brightness	ResNet, VGG and EfficientNet-B2	80/20, 10-fold Cross-Validation	Accuracy (87.15%), Precision (87.00%), Recall (87.00%), and F1 score (87.00%)
Yotsu et al. (2023) [85]	Leprosy, Buruli Ulcer, Mycetoma, Scabies, and Yaws	Images	Images resized to 224 × 224, Data augmentation and normalization	ResNet-50 and VGG-16, optimized by Stochastic Gradient Descent (SGD)	70/30	Accuracy (84.63%)
Barbieri et al. (2022) [12]	Leprosy and other skin diseases	Images, Numerical Data	Numeric data: normalization. Images: tuning strategy or freeze	Inception-V4, ResNet-50, Elastic-net Logistic Regression (LR), XGBoost (XGB) and Random Forest (RF)	Dataset split into 80% training, 20% test (80/20); 5-fold and 10-fold cross-validation	Accuracy (90.00%), Area Under Curve (AUC) (96.46%), Sensitivity (89.00%), and Specificity (91.00%)
Marçal et al. (2022) [14]	Paucibacillary or Multibacillary Leprosy	Numerical Data	Not Available	Decision Trees (DT)	Leave-one-out-cross-validation (LOOCV)	Accuracy (84.00%)
Steyve et al. (2022) [86]	Leprosy, Leishmaniasis, Buruli Ulcer	Images	OTSU thresholding and filters Canny, Sober, Gabor, and Robert	Support Vector Machine (SVM), SVM optimized by Black Hole Algorithm (BHO), K-Nearest Neighbors (KNN), DT	Not Available	Accuracy (96.00%), Specificity (94.00%), F-Score (89.00%), Recall (90.00%), and Sensitivity (92.00%)
De Souza et al. (2021) [87]	Paucibacillary or Multibacillary Leprosy	Numerical Data	Not Available	RF	10-fold cross-validation	Accuracy (92.38%), Sensitivity (93.97%), and Specificity (87.09%)
Tió-Coma et al. (2021) [76]	Leprosy	Numerical Data	Not Available	RF	80/20; LOOCV	Accuracy (87.50%), Sensitivity (100.0%), Specificity (80.0%), and AUC (96.70%)
Jaikishore et al. (2021) [88]	Leprosy, Eczema, and Measles	Images	Re-scaling to normalize the image, zoom in and zoom out, width shift, height shift, and rotation angle of 45°	MobileNet-V2, VGG16, Inception-V3, Xception	80/20	Accuracy (94.32%), F1 score (93.02%), Precision (93.53%), and Recall (92.76%)
Banerjee et al. (2020) [89]	Leprosy, Vitiligo, and Tinea versicolor	Images	Local Binary Pattern (LBP), Weber Local Descriptor (WLD), Gray-Level Co-Occurrence Matrix (GLCM), riLBP (rotation invariant LBP) and WLDRI (rotation invariant WLD)	GoogLeNet, MobileNet-V1, ResNet-152, DenseNet-121, ResNet-101 and SVM	80/20	Accuracy (91.38%)
Jin et al. (2020) [90]	Leprosy, Thalassemia, Hyperthyroidism, and Down’s syndrome	Images	OpenCV, Histogram of Oriented Gradients (HOG), Dlib library, ResNet50, VGG16	SVM Linear	80/20	Accuracy (93.30%)
Mondal et al. (2020) [91]	Leprosy, Tinea versicolor, and vitiligo	Images	Images cropped and centered a Region of Interest (ROI) manually, Global Contrast Normalization (GCN), Generative Adversarial Network (GAN), Wasserstein GAN with gradient penalty (WGAN-GP)	ResNet-101, DenseNet-169 and DenseNet-121	80/20	Accuracy (94.00%), Recall (90.00%), and F1 score (92.00%)
Casuayan and Devaraj (2020) [92]	Leprosy, Acne Vulgaris, Atopic Dermatitis, Keratosis Pilaris (Chicken Skin), Psoriasis and Warts	Images	Dull Razor Algorithm, GLCM, Sharpening filter, Median filter, Smoothing filter, Binary mask, Sobel Operator	ANN and SVM	70/30; 10-fold cross-validation	Precision (96.55%), and Recall (93.33%)
Amruta et al. (2019) [93]	Leprosy, Melanoma, Eczema	Images	Histogram equalization, Global Thresholding, Thresholding, GLCM	Iterative Dichotomiser 3 (ID3)	Not Available	Accuracy (87.00%)
Gama et al. (2019) [94]	Paucibacillary or Multibacillary Leprosy	Numerical Data	Not Available	RF	Not Available	Sensitivity (90.50%), and Specificity (92.50%)
Baweja and Parhar (2016) [95]	Leprosy	Images	Not Available	Inception-V3	80/20; Dataset into 50% positive and negative images	Accuracy (91.60%)
Pillai and Chouhan (2014) [96]	Leprosy	Numerical Data	Not Available	Sequential Minimal Optimization (SMO), LibSVM and Multilayer Perceptron (MLP)	80/20; Stratified 5–fold and 10-fold cross-validation	Accuracy (85.00%)
Yasir et al. (2014) [97]	Leprosy, Eczema, Acne, Psoriasis, Scabies, Foot ulcer, Vitiligo, Tinea Corporis, Pityriasis rosea	Images	Filters sharpening, median, smooth, binary mask, histogram, YCbCr algorithm, Sobel operator	Feed-Forward Back Propagation Network (FFBPN)	85/15; 10-fold cross-validation	Accuracy (90.00%)
Das et al. (2013) [98]	Leprosy, Tinea versicolor, Vitiligo	Images	LBP, GLCM, Discrete Cosine Transform (DCT), and Discrete Fourier Transform (DFT)	LibSVM	80/20	Accuracy (89.66%)
Pal et al. (2013) [99]	Leprosy, Tinea versicolor, Vitiligo	Images	Differential Excitation, WLD Histogram, WLD, WLDRI	SVM	80/20	Accuracy (86.78%)

## Data Availability

All relevant data are within this manuscript and its Supplementary Files.

## Supplementary Materials

The following supporting information can be downloaded at: https://www.mdpi.com/article/10.3390/jcm13010180/s1, Supplementary File S1: PRISMA 2020 Checklist; Supplementary File S2: Quality Assessment—Cabitza and Campagner’s checklist (2021)—Artificial Intelligence on Diagnostic Aid of Leprosy: A Systematic Literature Review.

## Abbreviations

The following abbreviations are used in this manuscript:

AI	Artificial Intelligence
ML	Machine Learning
DL	Deep Learning
CV	Computer Vision
NTD	Neglected Tropical Disease
CNN	Convolutional Neural Network
ANN	Artificial Neural Network
WHO	World Health Organization
PB	Paucibacillary
MB	Multibacillary
PGL-1	Phenolic glycolipid-I
ELISA	Enzyme-linked immunosorbent assay
PCR	Polymerase Chain Reaction
SVM	Support Vector Machine
RQ	Research Questions
QC	Quality Criteria
mR	Minor revision required
MR	Major revision required
ADAM	Adaptive Moment Estimation
SGD	Stochastic Gradient Descent
LR	Logistic Regression
XGB	XGBoost
RF	Random Fores
AUC	Area Under Curve
DT	Decision Trees
LOOCV	Leave-one-out-cross-validation
KNN	K-Nearest Neighbors
LBP	Local Binary Pattern
WLD	Weber Local Descriptor
GLCM	Gray-Level Co-Occurrence Matrix
riLBP	rotation invariant LBP
HOG	Histogram of Oriented Gradients
ROI	Region of Interest
GCN	Global Contrast Normalization
GAN	Generative Adversarial Network
ID3	Iterative Dichotomiser 3
SMO	Sequential Minimal Optimization
MLP	Multilayer Perceptron
FFBPN	Feed-Forward Back Propagation Network
DCT	Discrete Cosine Transform
DFT	Discrete Fourier Transform
RNA-Seq	RNA sequencing technique
RT-qPCR	Real-time Reverse Transcription Polymerase Chain Reaction
NGS	Next-generation sequencing
TNF	Tumor Necrosis Factor
IFN-y	Interferon-gamma
IL-4	Interleukin 4
IL-10	Interleukin 10
IgG	Immunoglobulin G
IgM	Immunoglobulin M
